# Comparative miRNA Expression Profiles in Individuals with Latent and Active Tuberculosis

**DOI:** 10.1371/journal.pone.0025832

**Published:** 2011-10-07

**Authors:** Chuan Wang, Shunyao Yang, Gang Sun, Xuying Tang, Shuihua Lu, Olivier Neyrolles, Qian Gao

**Affiliations:** 1 Key Laboratory of Medical Molecular Virology and Institute of Biomedical Sciences, Fudan University, Shanghai, China; 2 Respiratory Branch, Shanghai Public Health Clinical Center Affiliated to Fudan University, Shanghai, China; 3 Institut de Pharmacologie et de Biologie Structurale, Centre National de la Recherche Scientifique and Université de Toulouse, Toulouse, France; Charité-University Medicine Berlin, Germany

## Abstract

The mechanism of latent tuberculosis (TB) infection remains elusive. Several host factors that are involved in this complex process were previously identified. Micro RNAs (miRNAs) are endogenous ∼22 nt RNAs that play important regulatory roles in a wide range of biological processes. Several studies demonstrated the clinical usefulness of miRNAs as diagnostic or prognostic biomarkers in various malignancies and in a few nonmalignant diseases. To study the role of miRNAs in the transition from latent to active TB and to discover candidate biomarkers of this transition, we used human miRNA microarrays to probe the transcriptome of peripheral blood mononuclear cells (PBMCs) in patients with active TB, latent TB infection (LTBI), and healthy controls. Using the software package BRB Array Tools for data analyses, 17 miRNAs were differentially expressed between the three groups (*P*<0.01). Hierarchical clustering of the 17 miRNAs expression profiles showed that individuals with active TB clustered independently of individuals with LTBI or from healthy controls. Using the predicted target genes and previously published genome-wide transcriptional profiles, we constructed the regulatory networks of miRNAs that were differentially expressed between active TB and LTBI. The regulatory network revealed that several miRNAs, with previously established functions in hematopoietic cell differentiation and their target genes may be involved in the transition from latent to active TB. These results increase the understanding of the molecular basis of LTBI and confirm that some miRNAs may control gene expression of pathways that are important for the pathogenesis of this infectious disease.

## Introduction

Tuberculosis (TB), caused by *Mycobacterium tuberculosis* (MTB), remains a threat to global health. In 2008, TB accounted for nearly 1.8 million deaths worldwide, second only to the human immunodeficiency virus (HIV) as an infectious cause of death [1]. Approximately ∼5 to 10% of MTB-infected individuals develop active TB at some stage in their life [2]. The remaining ∼90 to 95% infected people remain asymptomatic, carrying so-called latent TB infection (LTBI), which is defined solely by the evidence of immunological sensitization to mycobacterial proteins (MTB-purified protein derivative, PPD) in the absence of clinical signs and symptoms of active disease [3]. The World Health Organization estimates that nearly one third of the world population is PPD+ [4]. This vast reservoir of LTBI-infected individuals is a constant source of disease caused by reactivation, especially in developing countries with large numbers of TB cases and a high TB incidence rate. The risk of TB reactivation among immunocompetent LTBI persons is estimated as 10% per lifetime. Impaired immunity such as HIV infection increases the risk to 10% per year and ∼50% per lifetime [5], [6].

The exact underlying mechanisms of LTBI and its transition to active TB remain elusive. LTBI rely on an equilibrium in which the host is able to control the infection but does not completely eradicate the bacteria [7]. Latency may depend upon the virulence of the MTB strain [8] and upon the host immune response. Some bacteria may escape attack from the innate or acquired immune system by blunting phagosome and lysosome fusion, nitric oxide production, antigen presentation, or other bactericidal processes from the host, and therefore survive in a phenotype called dormancy [9]. Immunosuppressants such as HIV infection or anti-tumor necrosis factor (TNF) treatment for rheumatoid arthritis may lead to the reactivation of these bacteria. Owing to the lack of a widely accepted animal model to study the pathogenesis of *M. tuberculosis*, population-based studies have been the best methods to reveal the complex biology of LTBI. Earlier studies which used whole-genome transcriptional profiling of peripheral blood mononuclear cells (PBMCs) [10] or whole blood cells [11] to characterize signatures of susceptibility or resistance to tuberculosis, described FcGR1B (CD64) as the most deregulated gene in individuals with active TB in Caucasian populations and African populations in which TB was highly endemic. Another study identified a whole-blood 393 transcript dominated by neutrophil-driven interferon (IFN)-inducible genes correlated with radiological extent of active TB and reverted to that of healthy controls after treatment [12]. By using intracellular flow cytometry staining of stimulated PBMCs with MTB-derived peptides, a recent report found a dominant TNF-α+ MTB–specific CD4+ T cell response that discriminated between latent infection and active disease [13]. Although these studies further our understanding of the fundamental biology of TB and offer leads for diagnosis and treatment options in the future, taken together they do not fully explain how the pathogen transitions from the latent stage to active TB.

MicroRNAs (miRNAs) are endogenous ∼22-nucleotide RNAs that play important regulatory roles in animals and plants by targeting mRNAs for cleavage or translational repression [14]. There are currently ∼1,000 human miRNAs sequences listed in the miRNA registry which may target about 60% of all mammalian genes [http://www.mirbase.org/], indicating that these small molecules play fundamental and global functions in human biology, including development [15], differentiation [16], apoptosis [17], metabolism [18], viral infection [19], and cancer [20]. MiRNAs also modulate the innate and adaptive immune responses to pathogens by affecting mammalian immune cell differentiation and the development of diseases of immunological origin [21], [22].The clinical application of miRNAs as diagnostic or prognostic biomarkers has already been demonstrated in various types of cancers [23], [24]. However, compared to their well-known role in cancer, the role of miRNAs in susceptibility and resistance to infectious disease, especially those of bacterial origin, is still poorly understood.

In the present study, we compared the miRNA expression profiles of PBMCs from patients with active TB, subjects with LTBI, and healthy controls in order to test the hypothesis that candidate miRNAs regulate the transition from LTBI to active TB. We used a miRNA microarray chip containing ∼960 probes to identify the differently expressed miRNAs, and performed real-time quantitative polymerase chain reaction (qPCR) for confirmation. The putative regulatory network of miRNAs that were differentially expressed in the samples from active TB and LTBI individuals was constructed based on predicted target genes and previously published genome-wide transcriptional profiles. Our study provides a greater understanding of the role of miRNAs-mediated regulated networks in the transition from latent to active TB.

## Results

### Expression profiles of miRNAs in PBMC from different groups

**Figure 1:**
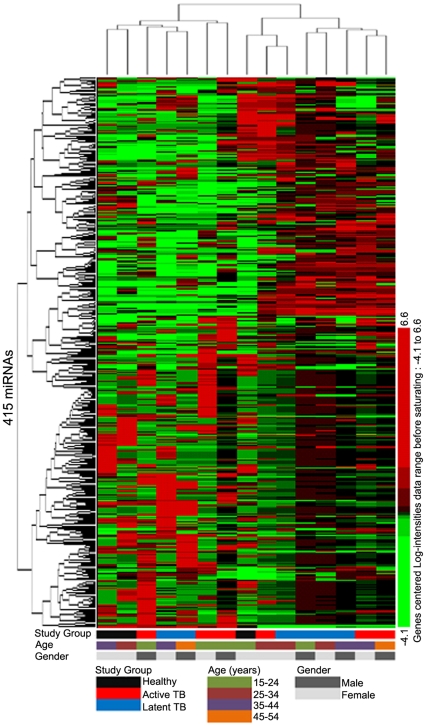
Unsupervised hierarchical clustering of the expression of 451 miRNAs. The tree was generated by unsupervised hierarchical clustering of PBMC miRNA profiles for patients with active TB, subjects with LTBI, and healthy controls. MiRNAs with 2-fold up- or down-expression compared with the median intensity across all samples and differential expression values greater than 10% of all samples were selected for unsupervised analysis (n = 415 miRNAs). Sample clusters can be compared with the clinical parameters displayed in blocks underneath each profile. A key is provided at the bottom of the figure. Subjects' clinical status is indicated as follows: patients with active TB are indicated by red rectangles; subjects with LTBI by blue rectangles; and healthy controls by black rectangles. Age is indicated as: subjects between 15–24 years old are indicated by olive green rectangles; subjects 25–34 years old by brown rectangles; subjects 35–44 years old by purple rectangles; and subjects 45–54 years old by khaki rectangles. Females are indicated by hoar rectangles and males by darkish rectangles.

We first determined whether the miRNA profile of patients with active TB was distinct from that of patients with LTBI and healthy controls. The demographic and clinical characteristics of all patients with active TB, LTBI, and the healthy controls are summarized in Table 1.We used a human miRNA microarray to perform the 955 miRNAs assay in PBMCs from 6 patients with active TB, 6 donors with LTBI, and 3 healthy persons for a total of 15 random selected biologically independent samples (Table S1). The microarray contains probes for 866 human and 89 human viral miRNAs represented in the Sanger miRBase (release 12.0, Sanger Institute, city, UK). Data analysis first consisted of unsupervised analyses of miRNA signatures in the dataset without a priori knowledge of the sample phenotypic classification [25]. After normalization and unsupervised filtering (see Materials and Methods), the data yielded a list of 415 miRNAs. The expression profiles of these 415 miRNAs were then subjected to unsupervised hierarchical clustering using the Pearson correlation with average linkage to create a condition tree (Fig. 1). The molecular classification obtained through hierarchical clustering was then compared with the phenotypic classification of the individuals from whom the samples were obtained. Two branches were obtained from the conditional tree that was generated: one branch was composed of 3 patients with active TB, 2 subjects with LTBI, and 2 healthy controls, while the other branch was composed of 3 patients with active TB, 4 subjects with LTBI, and 1 healthy control. This result showed that there was no clear distinction between the samples in regards to their clinical classification or age and gender, which indicates a high variability of whole miRNA profiles between individuals.

**Table 1:** Characteristics of study participants with active tuberculosis (TB), latent TB infection (LTBI) and healthy controls.

	Active TB			
Characteristic	Pre-treatment	Re-treatment	Latent TB infection	Healthy Controls
Total Number	28	1	29	18
Gender				
Female	9	1	17	16
Male	19	0	12	2
Ethnicity				
Han Chinese	28	1	29	18
Age, Mean Years ± SEM	38.2±3.8	47	40.8±2.0	35.1±2.8
Diseases Characteristics				
Pulmonary TB	26	1	Na	Na
Lymph node TB	1	0	Na	Na
Renal TB	1	0	Na	Na
TST				
Positive	Na		29	3
Negative	Na		0	15
IGRA				
Positive	24	1	29	0
Negative	4	0	0	18
HIV	-	-	-	-
HBV	-	-	-	-
HCV	-	-	-	-
Diabetes	-	-	-	-

NA, Not applicable; TST, Tuberculosis Skin Test; IGRA, Interferon-γ Release Assay; TB, Tuberculosis; HIV, Human immunodeficiency virus; HBV, hepatitis B virus; HCV, hepatitis C virus; SEM, standard error of the mean.

Subtle differences in gene expression among closely related subtypes may escape detection when using unsupervised clustering analysis. Therefore, we used BRB Arraytools to perform a F-test comparison by supervised clustering in an attempt to identify deregulated miRNAs between the different study groups [26]. We first differentiated the 3 groups by using a supervised learning algorithm to classify three or more groups (binary tree classification). Expression profiles were first classified into group A (Active TB) and group non-A (non-Active) (LTBI, Healthy) (node 1). The non-A group was then classified into LTBI and Healthy (node 2). Then, the same classification was used to generate node 3 (Healthy and Non Healthy), node 4 (Active TB and LTBI), and node 5 (Latent and Non Latent), and node 6 (Active TB and Healthy). By performing the 6 univariate tests (*P*<0.01) we identified a total of 38 miRNAs (Table S2 and Figure S1) that were statistically different (*P*<0.01) in at least one comparison. Next, we used Support vector machines (SMVs) as a prediction method to capture the differently expressed miRNAs among the 3 groups (Figure S1) [27]. Out of 10 miRNAs that differentiated node 1 (Active and Non Active) classification, 7 also differentiated node 4 (Active and LTBI) classification. These 7 miRNAs were then marked as the differentially expressed miRNAs between Active TB and LTBI. From the 6 univariate test results, a total of 17 miRNAs (Table S3) were identified using this prediction method (*P*<0.01).

Among these 17 miRNAs, 7 miRNAs (hsa-miR-130b*, hsa-miR-21*, hsa-miR-223, hsa-miR-302a, hsa-miR-424, hsa-miR-451, hsa-miR-486-5p) were differentially expressed between active TB and latent TB, 6 miRNAs were up-regulated in active TB patients, and only hsa-miR-130b* showed reduced gene expression level. 7 miRNAs (hsa-miR-144,hsa-miR-133a,hsa-miR-365, hsa-miR-424 ,hsa-miR-500, hsa-miR-661,hsa-miR-892b) had different gene expression levels between active TB and healthy controls; 4 of them (hsa-miR-144,hsa-miR-365 and hsa-miR-133a, hsa-miR-424) were up-regulated and 3 of them (hsa-miR-500, hsa-miR-661,hsa-miR-892b) were down-regulated in active TB patients. Five miRNAs (hsa-miR-130a*, hsa-miR-296-5p, hsa-miR-493*, hsa-miR-520d-3p, hsa-miR-661) had different expression levels between latent TB and healthy controls; all of them except hsa-miR-296-5p were up-regulated in healthy controls. Only hsa-miR-424 was up-regulated in comparisons of active TB with LTBI, as well as active TB with healthy controls. Hsa-miR-661 was up-regulated in comparisons of healthy controls with active TB, as well as healthy controls with LTBI.

**Figure 2:**
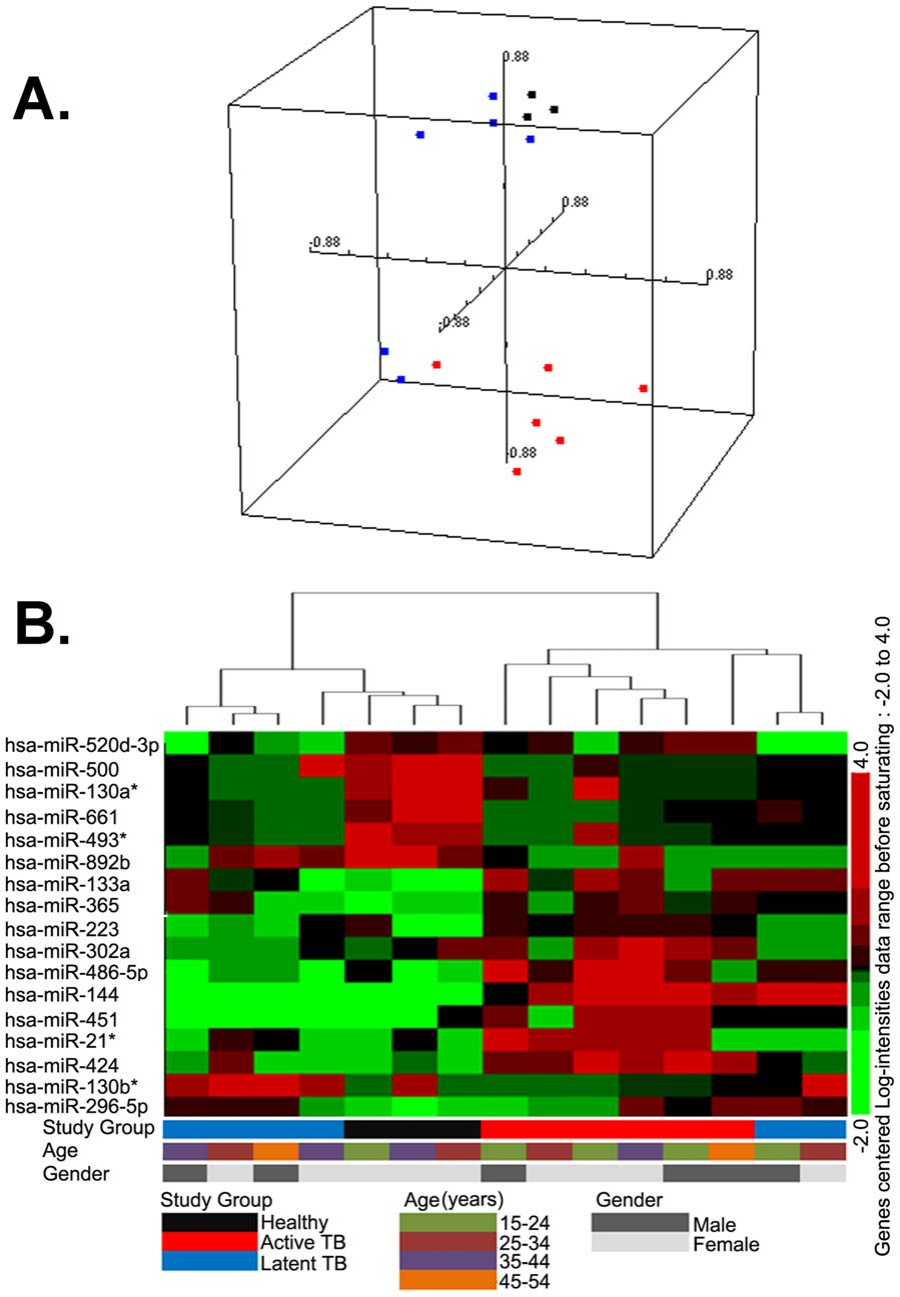
Multi-dimensional scaling of the relationships and unsupervised hierarchical clustering based on the 17 miRNAs expression pattern. A. Multi-dimensional scaling generated by BRB Arraytools of the relationships between the 15 samples based on the 17 miRNAs expression pattern. Red dot, active TB; Blue dot, latent TB; Black dot, healthy controls. B. The tree generated by unsupervised hierarchical clustering (Pearson's correlation, average linkage) of 15 samples (columns) and 17 miRNAs differentially expressed among the 3 groups predicted by using the SVM method. Sample clusters can be compared with the clinical parameters displayed in blocks underneath each profile. A key is provided at the bottom of the figure. Subjects' clinical status is indicated as follows: patients with active TB are indicated by red rectangles; subjects with LTBI by blue rectangles; and healthy controls by black rectangles. Age is indicated as: subjects between 15–24 years old are indicated by olive green rectangles; subjects 25–34 years old by brown rectangles; subjects 35–44 years old by purple rectangles; and subjects 45–54 years old by khaki rectangles. Females are indicated by hoar rectangles and males by darkish rectangles.

The multi-dimensional scaling generated by BRB Arraytools to visualize the relationships between the 15 samples based on the expression patterns of 17 miRNAs showed the active TB group were located in a different quadrant from the major group of non-active TB, which were composed by 4 latent TB and 3 healthy controls (Figure 2A). Only minor differences existed between latent and healthy controls. This revealed a discriminative pattern between active TB versus latent TB or healthy controls. Hierarchical clustering of the expression profiles of 17 miRNAs also showed that individuals with active TB clustered independently of individuals with LTBI or healthy controls (Figure 2B). This difference between active and non-active TB groups was mainly due to the induced expression of hsa-miR-365, hsa-miR-223 and hsa-miR-302a, hsa-miR-486-5p, hsa-miR-144 and hsa-miR-451, hsa-miR-21* and hsa-miR-424 in active TB patients. Interestingly, 5 of the miRNAs (hsa-miR-365, hsa-miR-223 and hsa-miR-144, hsa-miR-451, hsa-miR-424) were highly expressed in PBMCs and their expression in 3 groups was confirmed by qPCR. Meanwhile, the LTBI group was divided into 2 sub-clusters; 2/6 had an expression profile similar to the expression profile of the active TB group, and 4/6 clustered with healthy controls. This finding indicates there may be different stages of latency as it progresses to active disease Thus, by using 17 miRNAs, we identified a gene expression pattern or profile that discriminates between active TB versus healthy controls with or without *M. tuberculosis* infection.

**Figure 3:**
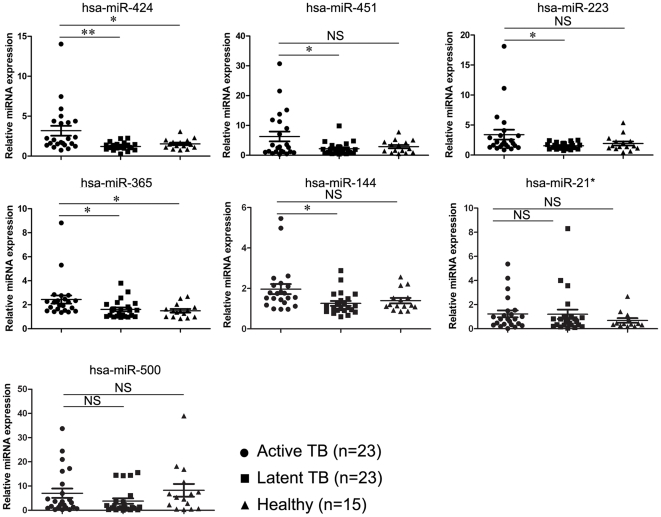
Quantitative PCR (qPCR) validation of miRNA expression levels in samples from the active tuberculosis (TB) group versus the latent tuberculosis infection (LTBI) groups. We confirmed the gene expression levels of miRNAs using real-time qPCR. We analyzed the expression of 7 miRNAs (hsa-miR-223, hsa-miR-365 hsa-miR-424, and hsa-miR-451, hsa-miR-144, hsa-miR-500 and hsa-miR-21*) selected from the microarray data by qPCR. The 2^−ΔΔCT^ method was used to normalize the relative gene expression data in the qPCR assay. U6 snRNA was set as the reference gene. The miRNA expression value in one subject with LTBI was normalized to 1. Statistical analysis was performed using the unpaired t-test. ** *P*<0.01, * *P*<0.05, NS: not significant.

To validate the microarray data, we used a SYBR-Green miRNA real-time qPCR analysis of miRNAs from PBMCs from 23 patients with active TB, 23 subjects with LTBI, and 15 healthy controls (Fig. 3). It should be noted that the expression intensities of some miRNAs (e.g. hsa-miR-500) in our microarray chip were just passed the threshold of detection; the low expressions of these miRNAs in PBMCs may lead to a poor reproducibility of RT PCR. Thus, in order to confirm our microarray data, we first classified all the 17 miRNAs into 2 groups based on expression values in PBMCs (reflected by mean of intensities in microarray). Those miRNA with mean of intensities in microarray >10 were classed to group 1, the rest miRNA Mean of intensities in microarray <10 were set as group 2 (Table S3).We picked 5 miRNAs from group 1; 3 miRNAs from group 2 representatively to perform the RT PCR confirmation. The expression profiles of the active TB and LTBI groups were statistically different for miRNAs from group 1: hsa-miR-144 (P<0.05), hsa-miR-424 (P<0.01), hsa-miR-451(P<0.05), hsa-miR-223 (P<0.05), and hsa-miR-365 (P<0.05). We observed that hsa-miR-424 and hsa-miR-365 also exhibited increased expression levels in samples from active TB versus healthy control groups (P<0.05). Our results were similar to those obtained in the microarray assay. Although most of the highly-expressed miRNAs in PBMCs showed significantly different expression levels between active TB and LTBI or healthy controls, not all microarray results could be confirmed by RT PCR. Microarray analysis and real-time PCR are two methods with different sensitivities and specificities, which might explain why target miRNAs at low levels could not always be detected by both methods [28].

### Construction of MiRNA-Gene-Network

**Figure 4:**
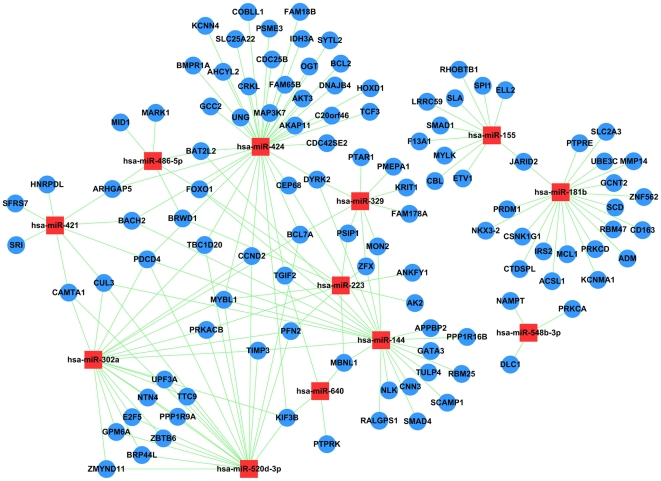
MiRNA-Gene-Network for the transition from latent to active tuberculosis. The MiRNA-Gene-Network was built using the gene expression data and the predicted interactions in the TargetScan miRNA database. The mRNA profile was obtained from previously published studies [10]. miRNA–mRNA modules obtained via population-based probabilistic learning was used to determine the interactions in the network (Materials and Methods). Circles represent genes and squares represent miRNAs; their relationship is represented by one edge. The center of the network represents the degree (i.e., the interaction of one miRNA with the genes around and the interaction of one gene with the miRNAs around. The key miRNAs and genes in the network always have the highest degrees.

The ultimate goal of our project is to test the hypothesis that the unique miRNA profiles and their target genes are associated with the progression of infection with *M. tuberculosis* from latent TB to active disease. Thus, we focused on miRNAs that are differentially expressed between active and latent TB. A previous study illustrated that there are alternated gene expression profiles in PBMCs between these 2 groups [10]. Based on our microarray data, several transcripts were negatively correlated with their predicted or validated miRNA targets, this revealed the existence of a miRNA-Gene regulatory network in the transition from latent to active TB. To identify the putative functional modules, we constructed the MiRNA-Gene-Network (Fig. 4) based on the data sets consisting of miRNA–target gene binding information and expression profiles of miRNAs and mRNAs [29]. We used the previously published mRNAs expression profiles from another study of samples from active TB and LTBI participants in the Gene Expression Omnibus (GEO) public database (www.ncbi.nlm.nih.gov/geo/query/acc.cgi?acc=GSE6112) [10]. We used this published report because their study, like the present study, used PBMCs in their RNA microarray assay.

We first performed an F-test between the Active TB and LTBI groups using a *P*-value<0.05 in order to select all the potential miRNAs expressed differentially. This test yielded a list of 17 miRNAs (Table 2) differentially expressed between these two groups. A total of 3010 target genes (Table S4) for these 17 miRNAs (taken from the TargetScan Database www.targetscan.org/) and their expression profiles were then imputed into the miRNA–mRNA modules designed before [30] (Materials & Methods). This method yielded a list of 12 miRNAs and 111 target genes with negatively correlated expression profiles (Table S5). There were no detectable correlations between hsa-miR-451, hsa-miR-342-5p and their target genes, which may be due to the small quantity of target genes for these 2 miRNAs in database (Table 2). Also we failed to retrieve the predicted or validated target genes for 3 miRNAs (hsa-miR-21*, hsa-miR-130b* and hsa-miR-550*) from the database. These 5 miRNAs were therefore absent in the network. Of the 12 miRNAs in the network, 3 were down-regulated in active TB, namely hsa-miR-155, hsa-miR-181b and hsa-548b-3p. The other 9 miRNAs exhibited increased expression in active TB. Hsa-miR-424 had the highest degree of regulation in the MiRNA-Gene-Network; 37 target genes were down-regulated in the mRNA expression profiles, followed by hsa-miR-144 with 23 target genes and hsa-miR-302a, hsa-miR-181b and hsa-520d-3p, both had 20 target genes in the network. Hsa-miR-548b-3p and hsa-miR-640 showed the minimum degree of regulation, with only 3 and 4 target genes included in the network.

**Table 2:** Differently expressed miRNAs in the samples from study participants with active tuberculosis (TB) versus latent tuberculosis infection (LTBI).

	Geom mean of intensities in						
miRNA	active TB (n = 6)	latent TB (n = 6)	Fold-change	Parametric p-value	Chromosomal location	No. Target Genes in TargetScan Database	No. Target Genes in the Network
hsa-miR-130b*	1.05	3.15	0.33	0.0004416	22q11.21	NA	NA
hsa-miR-223	9225.58	5243.09	1.76	0.0005922	Xq12	207	10
hsa-miR-424	49.49	15.18	3.26	0.0009967	Xq26.3	984	37
hsa-miR-302a	2.73	1.14	2.41	0.0042495	4q25	616	20
hsa-miR-21*	4.05	1.35	2.99	0.0046189	17q23.1	NA	NA
hsa-miR-520d-3p	3.25	1.47	2.21	0.0066037	19q13.42	616	20
hsa-miR-486-5p	105.78	31.62	3.35	0.0083258	8p11.21	111	6
hsa-miR-451	2514.81	596.91	4.21	0.0093827	17q11.2	14	0
hsa-miR-342-5p	37.32	87.84	0.42	0.0183201	14q32.2	65	0
hsa-miR-550*	2.36	1.18	2	0.0275742	7p14.3	NA	NA
hsa-miR-421	1.94	1.09	1.78	0.0281778	Xq13.2	276	8
hsa-miR-640	1.84	1.07	1.72	0.0289444	19p13.11	110	4
hsa-miR-144	27.22	3.39	8.04	0.0355072	17q11.2	661	23
hsa-miR-155	48.24	95.07	0.51	0.0438122	21q21.3	284	11
hsa-miR-181b	18.25	32.93	0.55	0.044274	1q32.1	904	20
hsa-miR-329	2.69	1.33	2.02	0.0446126	14q32.31	225	9
hsa-miR-548b-3p	1.05	2.1	0.5	0.045977	6q22.31	163	3

NA None of the target genes for these miRNAs were previously registered in the TargetScan database.

Regarding the 111 genes, most of them (78 in 111) were targeted by only one miRNA in the network (e.g. BCL2 was only targeted by hsa-miR-424). The rest 33 genes were regulated by multi-miRNAs: 14 genes were regulated by 2 miRNAs (e.g. CEP68 was targeted by hsa-miR-424 and hsa-miR-223), and 13 genes were regulated by 3 miRNAs.. MYBL1 (v-myb myeloblastosis viral oncogene homolog (avian)-like 1) and PDCD4 (programmed cell death 4) were regulated by 5 miRNAs (the highest degree of regulation in the network), followed by BCL7A (B-cell CLL/lymphoma 7A), CUL3 (cullin 3), FOXO1 (forkhead box O1), and KIF3B (kinesin family member 3B) which were regulated by 4 miRNAs in the network. Analysis of the Gene Ontology (GO), a database based on molecular functions (Fig S2A), and the Kyoto Encyclopedia of Genes and Genomes (KEGG), a regulatory pathway database (Fig. S2B) for these 111 genes showed that most encoded proteins with transcription regulator activity and protein binding functions were involved in cellular growth, movement, and proliferation, such as focal adhesion, MAPK signaling, Wnt signaling, insulin signaling, TGF-beta signaling, and chronic myeloid leukemia. Our results suggest that the miRNAs may regulate MTB infection by affecting the development of immune cells.

## Discussion

Our understanding the mechanisms of the transition from latent infection to active TB remains incomplete [3]. Multiple host factors are involved in this complex process. Herein, we focused on miRNAs, a class of ∼22-nucleotide short non-coding RNAs that play key roles in fundamental cellular processes. The miRNA registry currently contains ∼1,000 human miRNAs sequences, which target about 60% of mammalian genes. Our microarray chip contains nearly 90% of these miRNAs. The expression profile of these miRNAs showed high variability between individuals, and was independent of their age, gender, or clinical phenotype. However, we were able to distinguish the expression profile of the active TB group from the expression profile of the latent TB group using a 17-miRNA signature that was predicted by the SVM method. The molecular classification obtained through hierarchical clustering of these 17 miRNAs showed a discriminative pattern between active TB versus healthy controls with or without M. TB infection. Two LTBI subjects outlying the non active TB cluster and clustering together with active TB patients. A previous study identified a 393-transcript signature in whole blood cells that would discriminate between the different groups, and also reported that 10–25% of the patients with LTBI clustered with patients with active TB [12]. This may indicate there are different stages of LTBI, and LTBI is in fact a spectrum of responses to TB infection, ranging from individuals who have completely cleared the infection to individuals who are incubating actively replicating bacteria in the absence of clinical symptoms [3]. Because this complex process is associated with changes in the host's immune response, one would expect differential expression of some genes or miRNAs. Unfortunately, we currently lack a “gold standard” for discriminating the different stages of latent TB infection, which has made it very difficult to determine whether the expression of a genes or miRNAs could reflect the spectrum of responses to TB infection in different stages of latent infection.

We focused on the miRNAs that were differently expressed in active versus latent TB, in order to identify miRNAs that might be correlated with resistance and susceptibility of TB. We identified 17 miRNAs that were differentially expressed. The majority (12 out of 17 miRNAs) were up-regulated in patients with active TB. Among these miRNAs, hsa-miR-223, hsa-miR-424, and hsa-miR-451 and hsa-miR-144 were highly expressed in PBMCs compared to other miRNAs. Interestingly, these 4 miRNAs are also involved in the hematopoietic differentiation process. Previous studies using a miR-223-knockout mouse showed an increased proration of granulocytes, which are morphologically hypermature and hypersensitive to activating stimuli, and have more fungicidal activity [31]. Expression of miR-424 is induced during monocyte-macrophage differentiation, and miR-424 subsequently down-regulates expression of the transcription factor NFI-A78 [32]. Another study showed that miR-424 promotes monocytic differentiation through combinatorial regulation with miR-155, miR-222, and miR-503 [33]. MiR-451, with its cluster miR144, is required for erythroid differentiation and homeostasis [34]. The increased expression of these miRNAs in active TB may lead to the changes in immune cell profile and the alterations of the host immune response during MTB infection. Although we did not perform a comparative phenotypic assay of cellular composition in our samples, several studies found a related phenotype in active TB patients. For example, a previous study that used whole-genome transcriptional profiling revealed altered gene expression profiles in different cell types, such as macrophages and NK cells, in samples from active and latent TB [11]. Studies have also shown an increase in the proportion of CD14+CD16+ inflammatory monocytes and a decrease in the proportion of CD4+ T cells, CD8+ T cells, and B cells in blood cells of patients with active TB [12]. However, it is still unknown whether this alteration of cellular composition and related gene expression in active TB patients is regulated by miRNAs.

A previous study that used PBMCs to elucidate whole genome expression profile differences between latent and active TB found that 407 genes were upregulated and 364 genes were down regulated in active TB (RVM-T test with a *P*<0.05) [11]. Among these 771 genes, 111 were targeted by the 12 miRNAs differentially expressed in our microarray assay. It should be noted that 6/7 of affected genes during active TB are not targeted by our identified miRNAs, including some genes with highest degree of differential expression between active TB and latent TB such as CD64, LTF and RAB33A, these genes may be targeted by some miRNAs yet to be identified, also there may be other post-transcriptional regulation such as RNA binding proteins .The miRNA-Gene –Network for these 12 miRNAs and 111 genes reveals the high regulatory role of miR-424, which targets 37 genes in the network. Most of miR-424 target genes, such as BACH2, BCL2, BCL7A, and FOXO1, are involved in cellular differentiation and development. For example, the transcription factor BACH2, together with other factors such as BLIMP1 appears to constitute a transcriptional regulatory network for the differentiation of B cells to plasma cell [35]. The specific function of BCL7A has not yet been determined, however, it may play a role in T -cell lymphoma [36]. Both BACH2 and BCL7A have been targeted by multiple miRNAs in the network. Therefore, we hypothesize the depressed expression level of these 2 genes by multiple miRNAs such as hsa-miR-223 and hsa-miR-424 may lead to a disorder in the proportions of T cells and B cells in active TB patients, which may disturb the delicate balance of immune control in MTB infection. Bcl-2 is the founding member of the Bcl-2 family of apoptosis regulator proteins encoded by the BCL2 gene, which has been supported a role for decreased apoptosis in the pathogenesis of cancer [37]. Previous studies showed that apoptosis is an innate defense function of macrophages against MTB infection. One study showed that avirulent strains of mycobacteria could stimulate the macrophage to undergo apoptosis, which results in a ‘cellular corpse’ with an impermeable envelope that prevents bacteria from escaping. In contrast, virulent mycobacteria caused macrophage death by a process that proceeds to necrosis, which produces a permeable cell membrane that enables bacteria to escape and spread [38], [39]. Another study also assumed that virulent mycobacteria are able to inhibit apoptosis by altering the BCL2 pathway [40]. It is necessary to clarify whether the inhibited expression of BCL2 by miRNAs results in the alternative forms of cell death and induces the spreading of MTB in active TB patients.

Previous studies used whole-genome transcriptional profiling to understand the host response to MTB infection and found some potential signatures in the development of the disease [10], [ 11], [ and 12]. However, limitations of the microarray methods used in these studies led to the dismissal of some unknown transcripts such as miRNA. We report herein the changes in miRNAs expression profiles associated with the transition from latent to active TB. We identified specific miRNAs that were differentially expressed among active TB, latent TB and healthy controls. Our miRNAs microarray results show that these small non–coding RNAs may potentially be used to discriminate between active TB disease and latent TB infection. Our results increase the understanding of the molecular basis of LTBI, and suggest that miRNAs expression may play an important role in the pathogenesis of this infectious disease by controlling related gene expression. In addition, the putative miRNA targets provide new tools to better characterize LTBI. Further testing and validation are needed to determine whether miRNAs are useful markers, and whether they identify progression from LTBI to active disease and response to therapy.

## Materials and Methods

### Ethics statement

The study was reviewed and approved by the local ethics committee (Shanghai Public Health Clinical Centre). Written informed consent was obtained from participants prior to their enrollment in the study.

### Patient selection and PBMC isolation

Patients were recruited and enrolled at the Shanghai Public Health Clinical Centre (Shanghai, China) from December 2008 through May 2009. The demographic and clinical characteristics of the 29 patients with active TB, the 29 subjects with LTBI, and the 18 healthy controls are summarized in Table 1. The diagnosis of active TB was based on clinical presentation, chest radiography, and acid-fast stain of sputum smear. Lymph node TB was diagnosed by microscopy of lymph fragments from fine needle aspiration. Renal TB was diagnosed by microscopy of urinary analysis, type B ultrasonography, and computerized tomography. All of the patients were HIV negative, as diagnosed by the Livzon Anti-HIV1/2 EIA Kit (Livzon Pharmaceutical Group Inc., Guangdong, China). Additional tests were also performed to detect hepatitis B virus (HBV) and hepatitis C virus (HCV) using the Abbott AxSYM anti-HBsAg and HCV 3.0 antibody assay kit (Abbott Laboratories, Illinois) to exclude HBV- and HCV-positive patients (these two diseases are highly prevalent in China). Patients with a history of diabetes were also excluded because diabetes could increase the risk of active TB. Peripheral venous blood was drawn from study participants, prior to the initiation of any anti-TB treatment. Study participants with LTBI and healthy controls were recruited from the staff at the Shanghai Public Health Clinical Centre. Potential study participants were excluded if they had a prior history of TB or another infectious disease. Tuberculin skin testing (TST) and interferon-gamma release assay (IGRA) (T-SPOT®.*TB*, Oxford Immunuotec, Oxfordshire, U.K.) results were used to distinguish between the two groups. The LTBI group was TST-positive (TST>10 mm) and IGRA-positive while the healthy controls were TST-negative (TST<5 mm) and IGRA-negative. Three donors with TST-positive results IGRA-negative results were also included as healthy controls. The active TB group included patients with pulmonary TB (n = 27), renal TB (n = 1), and TB of the lymph node (N = 1); 28 of these patients were receiving TB treatment for the first time, and 1 was a case of retreatment TB. Patients with a positive sputum smear test result were diagnosed with pulmonary TB, and TST was not performed on them. There was no significant difference in age between the different groups (P-value = 0.412, one-way ANOVA test). However, there was a high proportion of males among patients with active TB and a high proportion of females among healthy controls., and the overall test for differences in gender was statistically significant (P-value = 0.0012, chi-square test).Peripheral venous blood (10 ml) was drawn from each subject and PBMCs were isolated on Ficoll gradients (GE Health Care, Little Chalfont, U.K.). PBMCs were immediately mixed with the miR VanaTM miRNA isolation kit Lysis Buffer (Ambion, Inc., Austin, Texas, USA) and frozen at −80°C until RNA was extracted. The separate RNA samples from a randomly chosen subgroup of 6 patients with active TB, 6 donors with LTBI, and 3 healthy controls (total of 15 biologically independent samples) were used in the microarray assay (see Table S1). The rest of the samples were used for real-time qPCR confirmation.

### RNA extraction and quantitation

Total RNA was extracted using the miR VanaTM miRNA isolation kit (Ambion Inc .Austin, Tx,USA) according to the manufacturer's instructions. RNA quantity and quality was assessed using the Nanodrop 2000 (Thermo Fisher Scientific, MA,USA) and Agilent 2100 bioanalyzer systems (Agilent Technologies). Samples with a RNA Integrity Number above 7 were used in the study.

### MiRNA microarray hybridization

MiRNA microarray assays were performed using the Agilent Human miRNA microarray platform (version 3, Agilent Technologies) at Shanghai Biochip Co., Ltd. (Shanghai, China). The microarray contains probes for 866 human and 89 human viral miRNAs from the Sanger miRBase (release 12.0, Sanger Institute, city, UK). Total RNA (100 ng) obtained from samples was labeled via Cy3 incorporation. Microarray slides were scanned by using the XDR Scan (PMT100, PMT5). Labeling and hybridization were performed according to the protocol in the Agilent miRNA microarray system.

### Microarray Data Submission

Microarray data submission for human arrays is MIAME-compliant. The raw data was submitted to the Gene Expression Omnibus (GEO) database, and is available under the following accession numbers: GSE29190; GPL10850; GSM72294–GSM722308.

### Computational analysis of miRNA microarray data

The microarray image information was converted into spot intensity values using the Scanner Control Software Rev. 7.0 (Agilent Technologies). The signal (after background subtraction) was exported directly into the GeneSpring GX9.0 software (Agilent Technologies) for normalization. The mean normalized signal from different groups was used for comparative expression analysis by using the software package BRB-ArrayTools developed by Dr. Richard Simon and the BRB-ArrayTools Development Team (http://linus.nci.nih.gov/BRB-ArrayTools.html). Data were filtered to exclude those miRNAs with expression values less than 10% in all samples. The remaining miRNAs were then filtered to select those having at least a 2-fold change in either direction from the miRNA's median intensity across all samples.After filtering, the data were then partitioned into 3 classes: Active TB, LTBI, and Healthy Control. Using the ‘class comparison’ multivariate permutation test and after averaging dye-swapped experiments, we identified genes that were differentially expressed between the different groups. Statistical analysis for differential expression was performed using random variance t-statistics for each miRNA. Hierarchical clustering was performed with the Pearson's correlation for differentially expressed miRNAs. The fold changes in the expression signals between different groups were calculated from the normalized values.

### Real-time qPCR analysis

miRNA real-time qPCR analysis was performed at CWBio.Co., Ltd. (Beijing, China). Briefly, the NEB *E. coli* poly (A) polymerase was first used to add poly (A) tail to total RNA. Complementary DNA was then synthesized by using the HiFi-MMLV reverse transcriptase (CWbio.Co., Ltd) and miRNA specific reverse transcription primers. SYBR Green (UltraSYBR Mixture, CWbio.Co Ltd) uptake in double-stranded DNA was measured using the Roche LightCycler® 480 Real-Time PCR System. We calculated 2^−ΔΔCT^ and used this statistic to determine relative gene expression. The reference gene was U6 snRNA.

### MiRNA-Gene network construction

To build the MiRNA-Gene-Network, we determined the relationship between miRNAs and genes by using their differential expression values, and the interactions between miRNA and genes in the TargetScan mRNA database. Briefly, published mRNAs expression profiles of active TB and latent TB were downloaded from the GEO public database (www.ncbi.nlm.nih.gov/geo/query/acc.cgi?acc=GSE6112).We used the RVM-T test (*P*<0.05) to identify the differently expressed genes and miRNAs from the gene expression profiles. The overlap between the genes whose expression was induced and the target genes whose expression was reduced by miRNA were then chosen to construct the network based on the two respective expression values by using the miRNA–mRNA modules via population-based probabilistic learning methods [26], [27]. The adjacency matrix of miRNA and genes A = [ai, j] was created by using the attribute relationships between genes and miRNAs, where ai,j represents the relationship weight between gene i and miRNA j. In the MiRNA-Gene-Network, circles represent genes and squares representing miRNAs; their relationship is represented by one edge. The center of the network represents the degree (i.e., the interaction of one miRNA with the genes around, or the interaction of one gene with the miRNAs around. The key miRNAs and genes in the network always have the highest degrees.

## Supporting Information

Figure S1 **Prediction of 17 miRNAs differentially expressed among different groups by using support vector machines (SMVs) method.** The gene expression profiles of three different groups of study participants (Active TB, Latent TB and Healthy group) were first differentiated using a supervised learning algorithm (binary tree classification). In Round 1, F-tests were first performed between node 1 (Active TB VS Non Active TB), node 3 (Healthy VS Non healthy), node 5 (Latent TB VS Non latent TB). The Round 2, F-tests were then performed between node 2 (Healthy VS Latent TB), node 4 (Active TB VS Latent TB), node 6 (Active TB VS Healthy). Finally, the miRNAs from both the Round 1 F test and the Round 2 F test were selected and marked as the miRNAs that were differentially expressed among different groups. miRNAs in red regarded those expressed differentially between Active TB and Latent TB; miRNAs in green regarded those expressed differentially between Active TB and Healthy; miRNAs in blue regarded those expressed differentially between Latent TB and Healthy; miRNAs in yellow regarded those expressed differentially among the 3 groups. Parametric p-value from all the F-test was <0.01.
(TIF)

Figure S2 **GO and KEGG annotations of 111 target genes in the network.** Enriched molecular functions of differentially expressed genes based on GO classifications (A) and KEGG pathways annotations (B) for genes targeted by miRNAs that were differently expressed between the active and latent TB groups. Functional annotation analysis was performed with the help of the Database for Annotation, Visualization and Integrated Discovery Bioinformatics Resources 2008 (http://david.abcc.ncifcrf.gov). Individual genes can be found under multiple GO/KEGG annotations. The percentages indicate the number of genes sharing a certain GO/KEGG term relative to the complete list of differentially expressed genes in both comparisons.
(TIF)

Table S1 **Characteristics of tuberculosis patients, latent TB infection and healthy control donors used for miRNA microarray.**
(DOC)

Table S2 **38 miRNAs differently expressed among active TB, latent TB, and healthy donors in at least one comparison by univariate tests (P<0.01).**
(XLS)

Table S3 **17 miRNAs differently expressed in microarray profiles among active TB, latent TB, and healthy donors by SVM prediction.**
(DOC)

Table S4 **3010 target genes (from TargetScan Database** www.targetscan.org/ **) of 17 miRNAs differently expressed between active tuberculosis (TB) and latent tuberculosis infection (LTBI).**
(XLS)

Table S5 **12 miRNAs and their 111 target genes in the network.**
(XLS)
