# Supplementary figures and images for: Comparative miRNA Expression Profiles in Individuals with Latent and Active Tuberculosis
Source: PLoS One. 2011 Oct 7;6(10):e25832. doi: 10.1371/journal.pone.0025832 (PMC3189221; doi:10.1371/journal.pone.0025832)

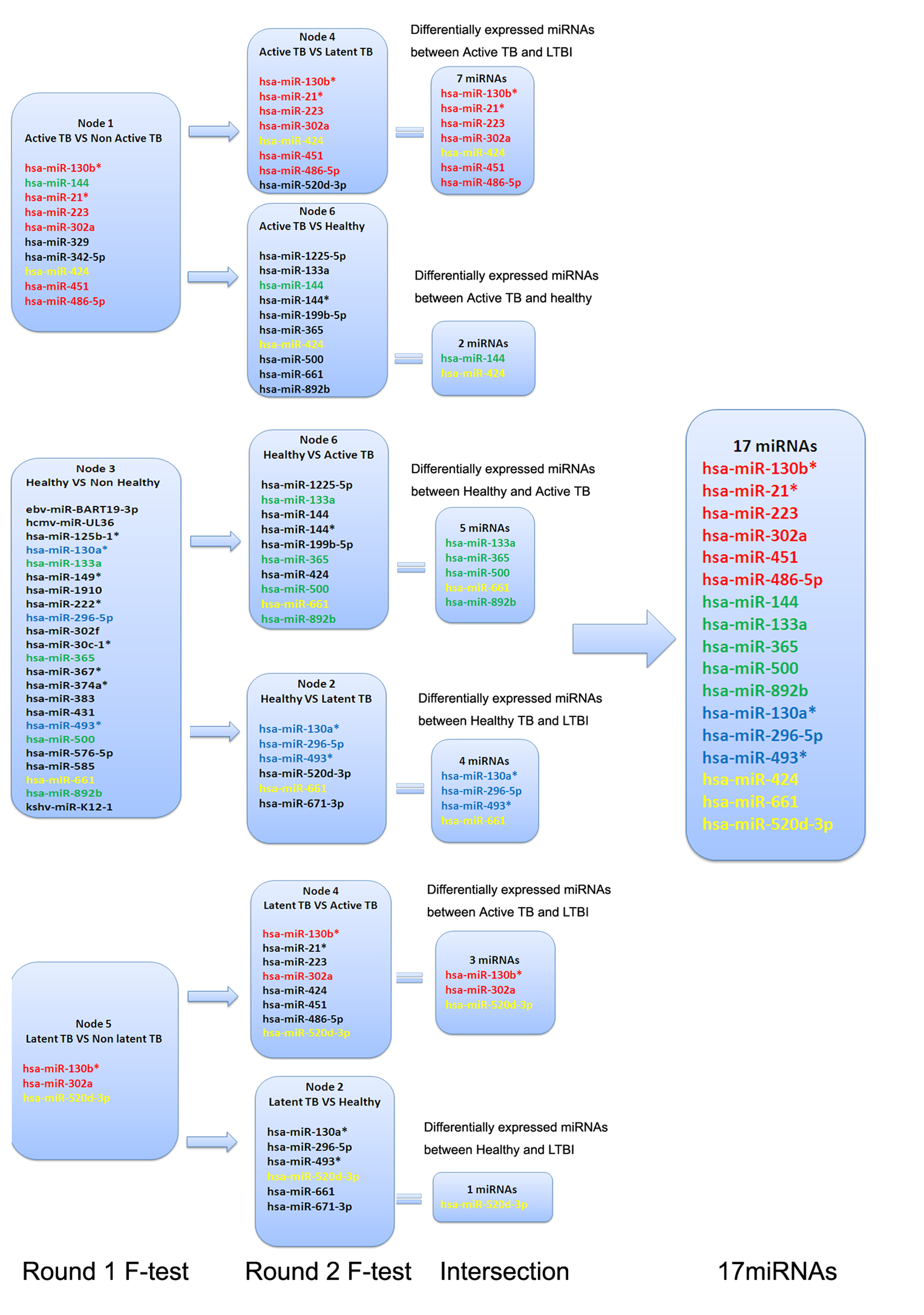

Supplement: Figure S1 — Prediction of 17 miRNAs differentially expressed among different groups by using support vector machines (SMVs) method. The gene expression profiles of three different groups of study participants (Active TB, Latent TB and Healthy group) were first differentiated using a supervised learning algorithm (binary tree classification). In Round 1, F-tests were first performed between node 1 (Active TB VS Non Active TB), node 3 (Healthy VS Non healthy), node 5 (Latent TB VS Non latent TB). The Round 2, F-tests were then performed between node 2 (Healthy VS Latent TB), node 4 (Active TB VS Latent TB), node 6 (Active TB VS Healthy). Finally, the miRNAs from both the Round 1 F test and the Round 2 F test were selected and marked as the miRNAs that were differentially expressed among different groups. miRNAs in red regarded those expressed differentially between Active TB and Latent TB; miRNAs in green regarded those expressed differentially between Active TB and Healthy; miRNAs in blue regarded those expressed differentially between Latent TB and Healthy; miRNAs in yellow regarded those expressed differentially among the 3 groups. Parametric p-value from all the F-test was <0.01. (TIF) [file pone.0025832.s001.tif]

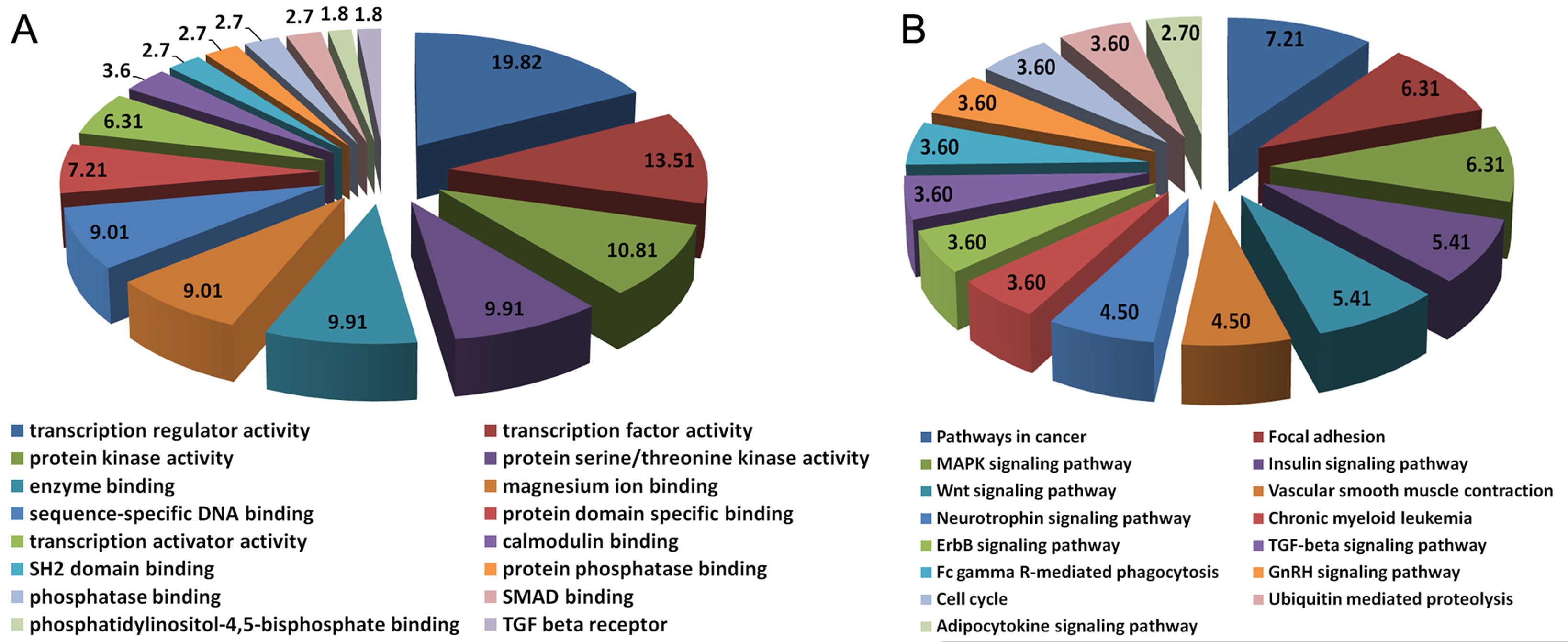

Supplement: Figure S2 — GO and KEGG annotations of 111 target genes in the network. Enriched molecular functions of differentially expressed genes based on GO classifications (A) and KEGG pathways annotations (B) for genes targeted by miRNAs that were differently expressed between the active and latent TB groups. Functional annotation analysis was performed with the help of the Database for Annotation, Visualization and Integrated Discovery Bioinformatics Resources 2008 (http://david.abcc.ncifcrf.gov). Individual genes can be found under multiple GO/KEGG annotations. The percentages indicate the number of genes sharing a certain GO/KEGG term relative to the complete list of differentially expressed genes in both comparisons. (TIF) [file pone.0025832.s002.tif]
