# Supplementary material for: Comparative miRNA Expression Profiles in Individuals with Latent and Active Tuberculosis
Source: PLoS One. 2011 Oct 7;6(10):e25832. doi: 10.1371/journal.pone.0025832 (PMC3189221; doi:10.1371/journal.pone.0025832)
Supplement: Table S1 — Characteristics of tuberculosis patients, latent TB infection and healthy control donors used for miRNA microarray. (DOC) [file pone.0025832.s003.doc]

## Table S1. Characteristics of tuberculosis patients, latent TB infection and healthy control donors used for miRNA microarray

| **Patient #** | **Gender^1^** | **Age (yr)** | **Diagnosis^2^** | **Pulmonary Radiology^3^** | **Sputum Smear** | **TST^4^** | **T-SPOT.TB** | **HBsAg** | **anti-HCV** | **anti-HIV** | **Diabetes** |
| --- | --- | --- | --- | --- | --- | --- | --- | --- | --- | --- | --- |
| **1** | **F** | **37** | **pTB, PT** | **+** | **+** | **na** | **+** | **-** | **-** | **-** | **-** |
| **2** | **F** | **28** | **pTB, PT** | **+** | **+** | **na** | **+** | **-** | **-** | **-** | **-** |
| **3** | **M** | **50** | **pTB, PT** | **+** | **+** | **na** | **+** | **-** | **-** | **-** | **-** |
| **4** | **F** | **15** | **pTB, PT** | **+** | **+** | **na** | **+** | **-** | **-** | **-** | **-** |
| **5** | **M** | **19** | **pTB, PT** | **+** | **+** | **na** | **+** | **-** | **-** | **-** | **-** |
| **6** | **M** | **18** | **pTB, PT** | **+** | **+** | **na** | **+** | **-** | **-** | **-** | **-** |
| **7** | **F** | **29** | **LTBI** | **na** | **na** | **+ +** | **+** | **-** | **-** | **-** | **-** |
| **8** | **M** | **36** | **LTBI** | **na** | **na** | **+ +** | **+** | **-** | **-** | **-** | **-** |
| **9** | **M** | **24** | **LTBI** | **na** | **na** | **+ +** | **+** | **-** | **-** | **-** | **-** |
| **10** | **F** | **34** | **LTBI** | **na** | **na** | **+ +** | **+** | **-** | **-** | **-** | **-** |
| **11** | **F** | **36** | **LTBI** | **na** | **na** | **+ +** | **+** | **-** | **-** | **-** | **-** |
| **12** | **M** | **53** | **LTBI** | **na** | **na** | **+ + +** | **+** | **-** | **-** | **-** | **-** |
| **13** | **F** | **39** | **Healthy** | **na** | **na** | **-** | **-** | **-** | **-** | **-** | **-** |
| **14** | **F** | **32** | **Healthy** | **na** | **na** | **-** | **-** | **-** | **-** | **-** | **-** |
| **15** | **F** | **24** | **Healthy** | **na** | **na** | **-** | **-** | **-** | **-** | **-** | **-** |

1: F Female, M Male.

2: pTB pulmonary tuberculosis, PT primary treatment.

3: na Not applicable

4: ++ + TST >15mm, ++ 10mm <TST <15mm, - TST < 5mm
