# Supplementary material for: Comparative miRNA Expression Profiles in Individuals with Latent and Active Tuberculosis
Source: PLoS One. 2011 Oct 7;6(10):e25832. doi: 10.1371/journal.pone.0025832 (PMC3189221; doi:10.1371/journal.pone.0025832)
Supplement: Table S3 — 17 miRNAs differently expressed in microarray profiles among active TB, latent TB, and healthy donors by SVM prediction. (DOC) [file pone.0025832.s005.doc]

**Table S**2. 17 miRNAs differently expressed in microarray profiles among active TB, latent TB ,and healthy donors by SVM prediction

|  | Geom mean of intensities in | | | Fold change in ^b^ | | |
| --- | --- | --- | --- | --- | --- | --- |
| miRNA | Active TB (n=6) | Latent TB (n=6) | Healthy (n=3) | Active TB / Latent TB | Active TB / Healthy | Latent TB / Healthy |
| Group 1^a^ |  |  |  |  |  |  |
| hsa-miR-144 | 27.22 | 3.39 | 1.67 |  | 16.3 |  |
| hsa-miR-424 | 49.49 | 15.18 | 11.92 | 3.26 | 4.15 |  |
| hsa-miR-451 | 2514.81 | 596.91 | 483.9 | 4.21 |  |  |
| hsa-miR-223 | 9225.58 | 5243.09 | 4573.52 | 1.76 |  |  |
| hsa-miR-365 | 22.34 | 18.61 | 9.31 |  | 2.4 |  |
| hsa-miR-296-5p | 10.24 | 12.83 | 5.12 |  |  | 2.51 |
| hsa-miR-486-5p | 105.78 | 31.62 | 23.63 | 3.35 |  |  |
| Group 2 |  |  |  |  |  |  |
| hsa-miR-130a* | 1.71 | 1.07 | 4.93 |  |  | -4.55 |
| hsa-miR-133a | 7.54 | 5.08 | 1.52 |  | 4.96 |  |
| hsa-miR-493* | 1.22 | 1.07 | 4.35 |  |  | -4 |
| hsa-miR-500 | 1.11 | 1.52 | 6.32 |  | -5.56 |  |
| hsa-miR-661 | 1.05 | 1.07 | 5.19 |  | -5 | -4.76 |
| hsa-miR-892b | 1.33 | 1.78 | 5.72 |  | -4.35 |  |
| hsa-miR-130b* | 1.05 | 3.15 | 1.51 | -3.03 |  |  |
| hsa-miR-21* | 4.05 | 1.35 | 1.27 | 3 |  |  |
| hsa-miR-302a | 2.73 | 1.14 | 1.68 | 2.39 |  |  |
| hsa-miR-520d-3p | 3.25 | 1.47 | 4.3 | 2.21 |  | -2.94 |

a Criteria set for different groups :

Gruop 1: Mean of intensities in microarray >10 in at least one group;

Group 2 Mean of intensities in microarray <10 in all groups

b Fold changes in comparison with Parametric p-value > 0.01 were not shown.
